# Retraction: Anthropogenic Radio-Frequency Electromagnetic Fields Elicit Neuropathic Pain in an Amputation Model

**DOI:** 10.1371/journal.pone.0191082

**Published:** 2018-01-05

**Authors:** The PLOS ONE Editors

After the publication of this article, readers raised concerns about several aspects of the methodology for this study and the conclusions drawn. Follow-up discussions with the authors and consultation with editorial board members identified the following concerns:

The analysis of skin temperature was found to be incorrect due to interference of the RFID-900-SC antenna with the ThermoWorks TW2 Thermometer, invalidating the measured skin temperatures during the reported experiments.

Dosimetry measurements in the paper were not obtained using appropriate equipment at the time the experiments were conducted, and thus the validity of the published data for RF-EMF exposure experiments has been called into question.

The authors agree that the temperature data in the published article are not valid, and they conducted additional experiments post-publication in an effort to address the dosimetry concern. However, the PLOS ONE editors determined that in light of the above issues the integrity of the published article is compromised, and the further work needed to address the dosimetry and temperature measurement errors and their impact on the conclusions drawn would necessitate full review as a new submission. For these reasons, we retract this published article.

The journal editors notified the article’s authors of this retraction. All co-authors BB, RGV, BRJ, EJ, and MRO expressed that they do not agree with the retraction.
